# Confidence in the face of risk: the Risk Assessment and Management Self-Efficacy Study (RAMSES)

**DOI:** 10.1192/pb.bp.112.040394

**Published:** 2014-04

**Authors:** Jaime Delgadillo, Omar Moreea, Hannah Outhwaite-Luke, Toby Dace, Brenda Nicholls, Georgina Ramseyer, Veronica Dale

**Affiliations:** 1 Primary Care Mental Health Service, Leeds Community Healthcare NHS Trust; 2 Department of Health Sciences, University of York

## Abstract

**Aims and method** To evaluate a comprehensive risk management programme. A Risk Assessment and Management Self-Efficacy Scale (RAMSES) was used to evaluate the impact of a clinical guideline and training course. Fifty-three psychological therapists were randomly allocated to training *v*. waiting list in a controlled, delayed-intervention design. Differences in mean self-efficacy scores between groups were examined using analysis of covariance (ANCOVA).

**Results** The RAMSES measure had adequate factor structure, internal consistency and construct validity. When adjusting for baseline scores and cluster design, the group exposed to training had a higher mean self-efficacy score than controls. Mean differences between groups were not significant after the control group received training, nor at 6 months’ follow-up.

**Clinical implications** Exposure to training and clinical guidelines can improve self-efficacy in risk assessment and management. An important advance put forward by this study is the specification of areas of competence in risk assessment and management, which can be measured using a psychometrically sound tool.

Approximately 32% of adults who die by suicide make contact with mental health services soon before death,^1^ and as many as 82% are reported to have made contact with their general practitioner (GP) during that time.^2^ Similarly, approximately 50% of adolescents and adults who disclose self-harm at emergency departments had contact with their GP in the previous month.^3^ Figures from a recent census indicate increased suicide rates from an age-adjusted rate of 7.9 to 11.8 deaths per 100 000 population between 2005-2007 and 2011-2012 in England.^4^ Suicide rates are comparatively higher for certain risk groups; notably, people under the age of 50, males (3:1 ratio of suicides compared with females), people who have had contact with mental health services, people with alcohol and drug dependence, psychiatric in-patients and prisoners.^5^ Rates of self-harm have been found to range between 2 and 7% in the UK general population aged 16-74 years.^6^ Although the rates of healthcare contact prior to suicide or self-harm vary among studies, there is general consensus that the communication of intent for such behaviours during contact remains low.^3,7-9^ The importance of assessing intent and the implications on future behaviours extends to other risk factors.^10^ For example, Munro & Rumgay^11^ highlighted the apparent lack of recognition of risk of violence during consultations with mental health professionals, suggesting that 11 out of 40 cases of homicide could have been identified through improved risk assessments. Others have also indicated that risks to self and others are associated with unplanned disengagement with primary care psychological therapy services.^12^

## Risk in mental health services: existing practices

Since the 1990s risk assessment and risk management have become ingrained within the standard practice of mental health services. In the past decade, English government policy has emphasised the notion of safety as essential to good practice.^3,13-16^ With this emphasis on safety and risk in mental health services, a number of standardised assessment tools, management models and training programmes have been developed.^14,17,18^ Risk assessment training has been suggested to be important in maintaining staff competence, although previous initiatives have mostly used non-validated satisfaction surveys and qualitative data as a means of evaluating training outcomes in uncontrolled study designs.^19-21^ Based on such data, it is unclear whether these training programmes indeed improve competence in this area. Furthermore, to our knowledge, there is no consensus about what would constitute areas of competence in risk assessment and management.

Training programmes in this area typically instruct clinicians on how to use standardised risk assessment tools. Alongside standardised tools, however, practitioners use their experience, knowledge and judgement to formulate risk assessment and management plans. It is this combination of multiple factors that leads practitioners towards a more structured clinical judgement approach to risk.^14^ However, healthcare professionals must feel competent and confident in employing what is essentially a complex, multifactorial approach to risk assessment and management. Qualitative research involving over 100 mental health staff, for example, suggests that confidence in professional judgements and practices is fundamental to safe and effective practice.^22^

## Self-efficacy in healthcare

Task- or work-specific confidence has often been conceptualised as related to the notion of ‘self-efficacy’. Following the theory developed by Bandura, self-efficacy refers to an individual’s perceived confidence in their capability to perform required actions to deal with prospective or future-oriented situations.^23,24^ Self-efficacy has been found to influence professionals’ behaviour, and enhanced self-efficacy leads to engaging more fully and effectively with tasks.^25^ A number of studies have demonstrated this effect, and specifically in relation to improving assessment practices in healthcare settings. For example, self-efficacy related to physical assessments was positively correlated to the frequency of these assessments in a study involving acute care nurse practitioners.^26^ In another study, a training programme designed to improve obstetrics and gynaecology residents’ attitudes and confidence in caring for patients with depression led to 3-month improvements in perceived self-efficacy and in the use of formal diagnostic tools.^27^ A lack of self-efficacy has also been suggested to be a potential barrier to healthcare professionals engaging in expected work behaviours,^28^ and has been shown to lead to avoidance or reduced effort in tasks, whereas higher self-efficacy leads to intensification of efforts.^29^ Furthermore, Bandura has argued that self-efficacy becomes more important when pursuing behaviour change in the face of adversity or obstacles, and that a high sense of self-efficacy in one domain does not necessarily transfer to other practice domains.^24^ Since self-efficacy is likely to be associated with health professionals’ behaviour, a deficit in the challenging domain of risk management may possibly result in poor effort or avoidance of these tasks, which in turn may result in neglect or poor response to risks and adverse events. In this sense, specifying the key domains and tasks pertinent to effective risk assessment and management is both relevant and important to mental healthcare, as is the consideration of self-efficacy within clinical training programmes.

**Table 1:** Demographic characteristics in Study 1

	Mental health teams *n* = 8	Substance misuse teams *n* = 4
Total participants, *n*	76	34
Females, *n* (%)	66 (87)	20 (59)
White British, *n* (%)	65 (86)	29 (85)
Age, years: median (range)	37 (23-63)	37 (24-55)
Experience, years: median (range)	10 (2-33)	10 (3-26)

This report describes the development and evaluation of a comprehensive risk assessment and management programme for mental health practitioners in primary care. The programme includes a clinical guideline, decision-making flowcharts, a training course and a structured questionnaire for use as a measure of self-efficacy in key areas of competence. The report presents two linked studies; the first study aimed to develop and validate the Risk Assessment and Management Self-Efficacy Scale (RAMSES) and the second study used this measure to evaluate the training programme and clinical guideline.

## Study 1: validation of the RAMSES measure

### Method

#### Participants and study design

Participating services included National Health Service (NHS) and voluntary-sector teams delivering evidence-based psychological interventions and addictions treatment and rehabilitation following national guidelines.^30-33^ A cross-sectional sample of mental health and substance misuse practitioners (*n* = 76, across 8 teams and *n* = 34, across 4 teams respectively) completed the study questionnaires using a secure electronic survey. Table 1 provides participants’ demographic characteristics.

#### Measures

RAMSES is a measure of task-specific self-efficacy, following the theory by Bandura.^24^ It has been modelled on other valid and reliable self-efficacy measures used to evaluate risk management training programmes in other healthcare settings.^34^ To our knowledge, there are no published comparable measures specific to risk management in mental healthcare. RAMSES contains a total of 18 items subdivided into three broad domains: assessment, management and referral (the actual questionnaire items are listed in Table 2). Each item is formulated as a specific task relating to one of the three broad domains or subscales. Respondents are prompted to rate their perceived self-efficacy on a Likert scale ranging from 0 (no confidence in ability to perform the task) to 10 (complete confidence in ability to perform the task). A composite self-efficacy score can be obtained by adding all item ratings and dividing the sum by 18.

This study included two comparative measures. The Addiction Counseling Self-Efficacy Scale (ACSES) measures self-efficacy in seven different areas: (1) clinical evaluation, (2) treatment planning, (3) referral, (4) service coordination, (5) counselling, (6) documentation and (7) professional and ethical responsibilities; all based on the national competencies for addiction counselling.^35^ This valid and reliable measure provided a criterion standard to evaluate the convergent validity of the RAMSES tool, that is, the degree to which it measures a theoretically similar construct (self-efficacy).

The Job Discrepancy and Satisfaction Scale (JDSS) addresses the extent to which practitioners are satisfied with their current working conditions including remuneration, supervision and autonomy. This instrument can be a useful tool to identify organisational factors that may need to be addressed to support and maintain a healthy workplace.^36^ This tool was included to examine discriminant validity, that is, the degree to which RAMSES measures a theoretically different construct (self-efficacy) than the construct measured by the JDSS.

#### Data analysis

Factor analysis was used to examine the underlying structure of the data-set of 18 items in the RAMSES questionnaire. We hypothesised that the questionnaire measures an underlying construct with three components: (1) the assessment of risks, (2) the management of risks in clinical practice, and (3) the referral process that may ensue when risks are detected. Following the general rule outlined by Bryant & Yarnold,^37^ we estimated a minimal sample size of 90 participants, based on a ratio of 5:1 between participants and scale items.

Principal components analysis (PCA) with varimax rotation and scree tests were used to perform factor analysis, which proceeds by extracting possible underlying factors and retains those which explain a large proportion of variance in the data. Conventional statistical tests were used to empirically evaluate the adequacy of the factor solution. The Kaiser-Meyer-Olkin (KMO) measure was calculated to determine whether any questionnaire items should be excluded from the final factor solution. In addition, Bartlett’s test of sphericity was used, where *P*<0.05 would be indicative of adequate factorability for the data-set as a whole.

After determining the factor structure of the questionnaire and deciding whether any items needed to be removed, we examined its validity and reliability using a series of conventional methods. Construct validity was tested by correlating RAMSES to another measure of self-efficacy (ACSES); and discriminant validity was assessed by correlating it to the JDSS scale which theoretically measures a different underlying construct (job satisfaction). These correlations were calculated using averaged scores (sum of items divided by total number of items), and were only conducted in the subgroup of substance misuse practitioners, since we could only find a comparable self-efficacy measure (ACSES) that was relevant and specific to the practice of that professional group. Parametric (Pearson’s) correlations between RAMSES scores and years of experience using the total sample were also performed, expecting that self-efficacy would positively correlate to experience. Conventional assumptions of normality and homoscedasticity were verified graphically and statistically (using Shapiro-Wilk test) before performing the above analyses. Finally, Cronbach’s α was used as a measure of internal consistency of questionnaire items, using α = 0.70 as a cut-off, with a higher number indicating acceptable reliability.^38^

## Study 1 results

### Factor analysis

Using Kaiser’s eigenvalue criteria (values >1.0), PCA indicated that three underlying factors accounted for 77.16% of variance in the data-set (factor 1 eigenvalue 11.50, accounting for 63.90% of variance; factor 2 eigenvalue 1.32, accounting for 7.35% of variance; factor 3 eigenvalue 1.07, accounting for 5.92% of variance). This was consistent with the scree test, which also indicated a three-factor solution based on eigenvalues above the cut-off of 1. After varimax rotation, factor 1 included items A4, B1, B2, B3, B4, B5 (eigenvalue 5.03, accounting for 27.92% of variance), and related to specific interventions to minimise risk. Factor 2 included items B6, B7, C1, C2, C3, C4 (eigenvalue 4.77, accounting for 26.50% of variance), and related to case management and referral in line with organisational policies. Factor 3 included items A1, A2, A3, A5, A6, B8 (eigenvalue 4.09, accounting for 22.73% of variance), and related to the assessment of risk. Table 2 presents the final rotated component matrix, displaying the correlations between the observed variables and the underlying factors. All factor loadings were above the minimal acceptable level of 0.40 on at least one factor, and we therefore decided to retain all 18 items in the final model. In addition, the adequacy of this set of variables for factor analysis was confirmed by Bartlett’s test of sphericity, which was non-significant (approximate χ^2^ = 2099.05, d.f. = 153, *P*<0.001). In line with these findings, the overall KMO measure of sampling adequacy was 0.92, indicating excellent factorability for the set of variables, and suggesting that a large amount of variance within the data can be explained by the underlying factor structure.

### Validity

Pearson’s correlations were performed with the subgroup of substance misuse practitioners (*n* = 34) who had completed both the ACSES and JDSS measures. RAMSES was highly correlated to ACSES which theoretically measures self-efficacy (*r* = 0.71; *P*<0.001), and was not correlated to JDSS which theoretically measures a different construct of job satisfaction (*r* = –0.33; *P* = 0.06). RAMSES scores were also positively correlated with years of experience in the whole sample (*n* = 109; *r* = 0.22; *P* = 0.02), indicative of a modest association between years of experience and self-reported self-efficacy levels.

### Reliability

Cronbach’s α for the RAMSES questionnaire using the whole sample (*n* = 110) was 0.96, indicative of high internal consistency based on a conventional cut-off of 0.70. Comparative α values between mental health and substance misuse practitioners (*n* = 76 and *n* = 34 respectively) were 0.97 and 0.95. These analyses all indicate a high level of internal consistency and reliability for the questionnaire in different professional groups.

**Table 2:** Principal components analysis after varimax rotation

How confident are you that you can:^a^	Factor 1: interventions	Factor 2: case management	Factor 3: risk assessment
A1. Use screening instruments to assess risk	0.026	0.218	**0.854**
A2. Interview people to elicit key information about risk factors	0.389	0.278	**0.783**
A3. Identify a person who is presenting risk to self	0.457	0.364	**0.672**
A4. Identify a person who is presenting risk to others	**0.659**	0.308	0.483
A5. Differentiate between people presenting high risk and low risk	0.414	0.511	**0.581**
A6. Synthesise relevant information in a formal or written risk assessment	0.369	0.318	**0.595**
B1. Use specific interventions focusing on risks of self-harm or self-neglect	**0.722**	0.330	0.343
B2. Help people to minimise the severity of risk to self	**0.771**	0.320	0.398
B3. Use specific interventions focusing on risks of harm to (or neglect of) others	**0.898**	0.288	0.177
B4. Help people to minimise the severity of risk to others	**0.884**	0.280	0.152
B5. Develop rapport with people who present significant risks	**0.491**	0.477	0.473
B6. Manage risks in line with organisational confidentiality policies	0.286	**0.749**	0.293
B7. Use strategies to avoid malpractice liability or disciplinary action	0.286	**0.764**	0.265
B8. Develop a formal or written risk management plan	0.466	0.425	**0.488**
C1. Appropriately judge whether or not a person should be referred to an external service or professional on the basis of risk	0.309	**0.694**	0.472
C2. Identify an appropriate service to refer someone on the basis of risk	0.171	**0.782**	0.375
C3. Successfully refer and engage a person with an appropriate service	0.360	**0.758**	0.217
C4. Motivate a person to successfully self-refer to an appropriate service	0.584	**0.683**	0.117

Bold indicates items that load highly on each of the factors.

a. RAMSES questionnaire, from 0, ‘Not at all confident’ to 10, ‘Highly confident’.

## Study 2: evaluation of training and clinical guideline

### Method

#### Risk management programme

The intervention in this study was a specialist training programme which follows the structure of a clinical guideline on the assessment and management of risks of harm to self or others^39^ and was based on an adaptation of the threshold model for risk assessment.^40^ The threshold model is based on research evidence indicating that the risk of suicide is influenced by a combination of predisposing factors (genetic factors such as family history, biological factors such as serotonin dysfunction, psychosocial factors such as history of childhood abuse, environmental factors such as social isolation, etc.), current trigger events (e.g. social, financial or family crisis, substance misuse), and protective factors (lack of access to methods of lethal self-harm, current support networks, personal values and attitudes towards suicide or self-harm, etc.).^41^ Following assessment, preventive measures for high-risk patients may include psychotherapeutic interventions, the negotiation of a risk management plan, and liaison with other professionals.^39^ The threshold model for suicidal risk outlines key risk assessment and management practices including: detection of major depression, thorough assessment of high-risk patients and suicide risk, prescription of adequate antidepressant medication, regular education of health professionals regarding risk, reducing availability of self-harm methods, managing substance misuse, and identification of family members at risk.^41^ The risk guideline and training programme contained detailed information on: (a) how to identify people at increased risk of self-harm, suicide or violence; (b) decision rules for risk management and appropriate interagency liaison; and (c) guidelines on defensible practice and clinical record-keeping. Participants were exposed to 2-hour workshops covering the above topics, and were provided with copies of the clinical guideline and decision-making flowcharts.

#### Participants and study design

A total of 53 mental health practitioners participated in risk management training, and completed baseline and follow-up measures. Demographic characteristics for this subsample are in Table 3. Participants were randomly allocated to cohort A (immediate training) and cohort B (waiting list control group with delayed training after 6 weeks). Cluster randomisation was used, grouping participants according to their teams to minimise potential contamination of the control group through routine peer discussions. All participants were asked to complete outcome measures at four time points: (1) pre-training; (2) after training cohort A; (3) after training cohort B; and (4) at 6 months’ follow-up.

**Table 3:** Demographic characteristics in Study 2

	Cohort A teams *n* = 3	Cohort B teams *n* = 4
Total participants, *n*	18	35
Females, *n* (%)	16 (89)	29 (83)
White British, *n* (%)	17 (94)	31 (89)
Age, years: median (range)	39 (24-59)	36 (26-63)
Experience, years: median (range)	13 (2-33)	11 (2-23)

#### Measures

The RAMSES questionnaire was used as the primary outcome measure.

#### Data analysis

Given the cluster randomisation design, the data were summarised for each cluster (7 clusters) and average RAMSES scores for each cluster were calculated at different time points. This is a methodologically robust alternative when analysing a relatively small number of clusters.^42,43^ To determine the effects of training, analysis of covariance (ANCOVA) was used with the post-intervention RAMSES score (time 2) as the dependent variable. A grouping variable (cohort) was entered as a fixed factor, a clustering variable (team) was entered as a random factor, and the pre-training RAMSES score was entered as a covariate to adjust for baseline self-efficacy measures. The analyses were weighted for the total number of participants in each cluster who responded at time point 2. Two further weighted ANCOVA models were used to compare group differences in mean self-efficacy scores at different time points, with time 3 and time 4 measures as the dependent variables. Model checking was performed by assessing residual plots to ensure all models fit the data. Finally, paired *t*-tests were used to compare pre-training *v*. 6 months’ follow-up scores for each cohort separately. Conventional assumptions of normality and equality of variances were tested prior to performing *t*-tests.

**Fig 1:**
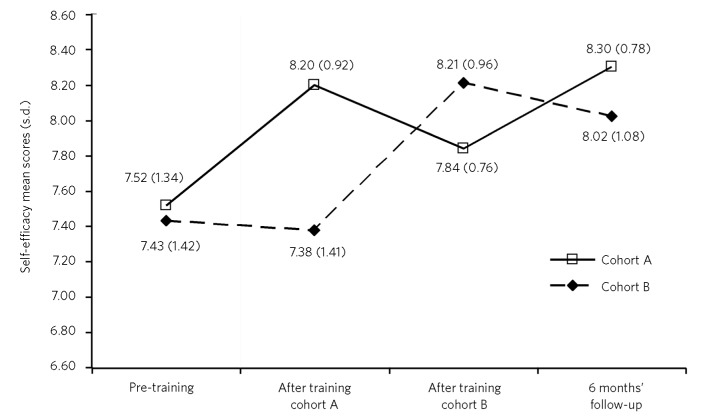
Mean (unadjusted) RAMSES scores at different time points in delayed intervention design.

## Study 2 results

Figure 1 displays mean self-efficacy scores (and standard deviations) for both cohorts at different measurement points. The lines for both cohorts generally indicate a trend of improvement in self-efficacy scores from baseline to 6 months’ follow-up, although the magnitude of change is modest given the fairly high entry-level scores before training. The chart shows that the wider difference in self-efficacy scores between groups is observed after cohort A had been trained, resulting in a higher mean score; cohort A 8.20 (s.d. = 0.92) *v*. cohort B 7.38 (s.d. = 1.41). Table 4 displays the results of the ANCOVA model, with adjusted mean scores for both cohorts at different time points. Adjusted mean differences were statistically significant at time 2, after training cohort A; 0.73 (95% CI 0.18 to 1.28), *P* = 0.021. Adjusted mean differences between groups were not statistically significant after cohort B was trained (0.46; 95% CI –0.74 to 1.65; *P* = 0.31), nor at 6 months’ follow-up (0.28; 95% CI –2.54 to 3.11; *P* = 0.71). Finally, pre-training and 6 months’ follow-up mean scores were significantly different for both cohort A (mean difference –0.63; 95% CI –1.13 to –0.12; *P* = 0.02) and cohort B (mean difference –0.34; 95% CI –0.67 to –0.02; *P* = 0.04) in an intention-to-treat analysis. Overall, these results indicate that increased self-efficacy was associated with exposure to training plus clinical guidelines, and these gains were sustained at 6 months’ follow-up.

**Table 4:** ANCOVA: RAMSES adjusted estimates^a^

Time point	Intervention estimate (s.e.)	Control estimate (s.e.)	Mean difference (95% CI)	*P*
Time 2: after training cohort A	8.143 (0.158)^b^	7.413 (0.117)^d^	0.730 (0.182 to 1.278)	0.021
Time 3: after training cohort B	7.781 (0.315)^c^	8.236 (0.204)^d^	0.455 (–0.742 to 1.652)	0.313
Time 4: at 6 months’ follow-up	8.303 (0.562)^c^	8.021 (0.339)^b^	0.282 (–2.542 to 3.105)	0.710

a. Estimates adjusted for baseline measures (time 1), weighted by number of responders at each time point. There were 6 weeks between time 1 and time 2; 6 weeks between time 2 and time 3; 6 months between time 3 and time 4.

b. Number of clusters = 3.

c. Number of clusters = 2.

d. Number of clusters = 4.

## Discussion

### Main findings

The RAMSES questionnaire was found to have a robust factor structure consisting of three components: clinical interventions, case management and assessment. Evidence of adequate internal consistency, construct and discriminant validity was also described for this measure. Limitations in the validation of the questionnaire related to the relatively small sample; particularly the low response rate for the substance misuse treatment subgroup which provided comparative data to assess construct and discriminant validity. Although the overall sample size was sufficient for psychometric testing according to conventional guidelines,^37^ the small sample size may have been influenced by response bias. Further validation of the measure in larger samples and in different clinical settings (e.g. social workers, forensic and hospital settings) would be advisable. Future validation studies could also consider the rotation of questionnaire items or the addition of reverse-keyed questions to control for acquiescence response bias.

This study presents evidence that training and development of clinical guidelines can improve mental health practitioners’ confidence in assessing and managing clinical risks. These data indicate that such gains in self-efficacy can be sustained for at least 6 months after training. Although statistically significant, observed improvements in self-efficacy measures were fairly modest. This was likely to be influenced by the high baseline measures in this professional group which led to ceiling effects, possibly owing to the large number of therapists with several years of experience. Our correlation analysis indicated modest positive associations between self-efficacy and years of experience; therefore, differences in mean years of experience between cohorts possibly account for the higher baseline self-efficacy mean in cohort A.

### Limitations

Some limitations in this trial concerned the small number of clusters (7), which required data analysis using summary measures for cluster means as proposed by Kerry & Bland.^42^ An important limitation is that this study did not investigate associations between changes in self-efficacy and any actuarial data such as measurable changes in practice or risk incidents. There is also the possibility that self-efficacy may not be a strong predictor of mental health practitioners’ behaviour change or improved outcomes. It is possible, for example, that overconfidence could actually result in inaccurate or complacent risk assessment, so the sensitivity and specificity of confident practitioners’ identification of high-risk cases is yet to be determined. In addition, estimating the predictive accuracy of clinicians’ risk detection and management is complicated by the relative low incidence rate of high-risk events at the level of individual mental health practitioners; for example, three-quarters of all those dying by suicide in the UK have not had contact with mental health services in the year before death.^16^ A further limitation of this study concerns the broad definition of clinical risk used in the RAMSES measure. This may not be specific enough to discriminate between acute or immediate risk, and lifetime risk or risk in relation to particular diagnoses or illnesses, and the presentation and subsequent management of these risks would potentially differ in clinical practice.

### Implications for practice and research

Mental health practitioners have a crucial role to play in the identification and prevention of serious risk incidents. This is clear from the detection point of view since increasing numbers of people are accessing mental health interventions in primary care services in England,^44,45^ and also from an intervention point of view since some psychological interventions (e.g. dialectical behaviour therapy, behaviour therapy for those who attempt suicide) are likely to help to increase treatment retention and to reduce the frequency of suicidal ideation and attempts.^46,47^ An important advance put forward by this study is the specification of areas of competence in risk assessment and management, which can be measured using a psychometrically sound tool. The RAMSES tool may be of relevance to mandatory training schemes for mental health specialists and other professionals whose role involves managing risk.

The present study adds to the increasing body of evidence demonstrating that training programmes can improve self-efficacy of healthcare staff.^34,48-51^ We argue that self-efficacy is important insofar as it reflects perceived confidence and competence on key aspects of clinical practice. Self-efficacy is closely related to the constructs of perceived control and perceived behavioural control; together, these aspects of self-perception are likely to predict intentions and actual behaviours.^52^ Bandura’s theory^24,53^ posits that task-specific behaviours can be increased by enhancing self-efficacy through vicarious learning, persuasion and enactive performance. However, the state of the evidence is still inconclusive with regard to impact on patient outcomes.^54^ It is still too early to assert that increasing self-efficacy will necessarily result in sustained changes in risk management practice or tangible outcomes such as net reductions in risk incidents in a given treatment population. Next steps in the advancement of knowledge in this field would require the collection of guideline-matched performance data from risk management plans as a means of evaluating behaviour change following exposure to clinical guidelines and training. The role of clinical supervision may also be a relevant focus of attention. For example, actuarial data on risk management practices and risk incidents may be collected as part of clinical supervision, which could be correlated with self-efficacy measures to explore the predictive value of this construct.

In conclusion, this study demonstrates that evidence-based risk training can enhance mental health professionals’ self-efficacy, and these gains are sustained for at least 6 months. We propose that comprehensive programmes of risk assessment and management training such as the one described here can be potentially helpful to other groups of professionals who come into contact with vulnerable individuals, for example health visitors, social workers, GPs, forensic and criminal justice professionals.
